# Report from the 2016 CrossConnects workshop: improving data mobility & management for bioinformatics

**DOI:** 10.1186/s40793-017-0297-4

**Published:** 2017-12-19

**Authors:** Kathryn Petersen Mace, Daniel Jacobson, Brooklin Gore, Lauren Rotman, Jennifer Schopf, Mary Hester, Predrag Radulovic, William Barnett

**Affiliations:** 1 Lawrence Berkeley National Laboratory, 1 Cyclotron Road, M/S 59R3103, Berkeley, CA 94720 USA; 2 Oak Ridge National Laboratory, Oak Ridge, USA; 3 Indiana University, Bloomington, USA; 4 SURFnet, Utrecht, Netherlands

**Keywords:** Genome sequencing, Metagenomics, Precision medicine, Bioinformatics, Data management, Data access, Data structure, Machine learning, National initiatives, Compliance, Shared datasets

## Abstract

Due to significant declines in the price of genome sequencing technology, the bioinformatics sciences are experiencing a massive upswing in data generation resulting in an increasing need for data distribution and access. The sheer number of biological areas of study, many of which benefit from the scientific breakthroughs of one another, are adding to the increase of shared data usage. The need for effective data management, analysis, and access are becoming more critical. While there are commonalities facing both precision medicine and metagenomics, each area has its own unique challenges and needs. A workshop was held in April 2016 at Lawrence Berkeley National Laboratory that brought together scientists from both fields, along with experts in computing and networking. Presenters and attendees discussed current research and pressing data issues facing the bioinformatics field today and in the near future.

## Introduction

The fourth workshop in the CrossConnects Workshop series was held April 12–13, 2016 at Lawrence Berkeley National Laboratory, titled, *Improving Data Mobility & Management for Bioinformatics*. The two-day workshop covered two primary focus areas in bioinformatics: Precision Medicine and Metagenomics.

During the workshop, participants identified data management as a continually growing and pressing issue, even more so than data mobility or data transmission speeds. Given data from The National Human Genome Research Institute (NHGRI) studies showing an exponential drop in the cost of genomic sequencing, which exceed Moore’s Law at an increasing rate [1], both data management and data mobility were noted as critical topics for successful bioinformatics research.

Data management and access issues were repeatedly mentioned throughout the workshop discussions. It was clear that many current data management solutions are in the early stages and will require additional and continued community effort in order to store, structure, and share data in efficient ways for collaborators around the world. It is important to realize that recent advances in technology are enabling discoveries at a rate that biological scientists and technologists are racing to keep up with. The workshop represented the forefront of thinking on these issues and highlighted the need to accelerate data solutions.

Bioinformatic sciences are dealing with their own set of unique challenges that must be addressed by the community and affiliated technologists. For example, in some cases scientific discoveries in the field are leading to breakthroughs that make prior research obsolete--the prior research having been published only 12 months ago or less. This reality, combined with the prevalence of DNA/RNA sequence data generation in numerous, widely dispersed geographic locations, makes the current state of bioinformatic sciences fundamentally different from other compute-intensive scientific disciplines (e.g., High Energy Physics, where massive amounts of data generation happens at a few large facilities, but is shared widely and analyzed on different computing ecosystems worldwide). These aspects, combined with privacy requirements around human health information, must be considered when devising sustainable data management and mobility solutions for the various branches of the bioinformatics community.

There are multiple national initiatives in progress, one of which was announced after the workshop, that are focusing on many of the topics discussed: The White House Precision Medicine Initiative (now ‘All of Us’), the Department of Energy (DOE) Office of Science Exascale Program, the National Cancer Moonshot, and the White House National Microbiome Initiative, announced May 13, 2016. These three White House Initiatives are discussed in the respective sections below. The DOE Exascale Program is currently collecting computational, software, and networking requirements from scientists in all major scientific disciplines, including Biological Research. The requirements that are being collected will shape the next generation of an exascale ecosystem “needed to support the forefront of scientific research through 2025” [2]. One workshop speaker stated that due to technological advances, this is the most exciting time he has been involved in bioinformatics research.

## Background & motivation

### Precision medicine

For several years, scientists and researchers have been working to advance the understanding of Precision Medicine, the customization of medical practices and treatments for individual patients and its techniques for the betterment of human health. As discussed in the workshop, 90% of what affects our health is not determined by medical care, but rather our environment, socio-economic circumstances, lifestyle choices, and genetics [3]. In January 2015, U.S. President Obama highlighted the Precision Medicine effort and called the country’s attention to its importance during his State of the Union Address when he announced the launch of The Precision Medicine Initiative. The Initiative is detailed on the White House website with the following mission statement:

*To enable a new era of medicine through research, technology, and policies that empower patients, researchers, and providers to work together toward development of individualized care* [4]*.*

The White House allocated a total of $215 million to the Department of Health and Human Services (DHHS) in support of the initiative in 2016. Also part of President Obama’s State of the Union Address was the National Cancer Moonshot initiative, a collaborative effort to end cancer. On February 1, 2016, the White House announced a $1 billion initiative to jumpstart the program and established “a new Cancer Moonshot Task Force – to be led by the Vice President – to focus on making the most of Federal investments, targeted incentives, private sector efforts from industry and philanthropy, patient engagement initiatives, and other mechanisms to support cancer research and enable progress in treatment and care” [5]. The multiple federal initiatives bring together experts in a variety of fields in order to work toward the common goal of improving the quality of life for all of us. This requires collaboration between experts around the world and amplifies the reliance on dependable data sharing practices.

The move toward Precision Medicine in healthcare models is accelerating the need for computational infrastructure to support collaborations among a wide range of professionals. The infrastructure must support the secure exchange of data between disparate groups. Currently, the biggest barrier to this is uncertainty about regulations covering access to data collected from patients and research participants, namely Protected Health Information (PHI), The Federal Information Security Management Act (FISMA), and The Health Insurance Portability and Accountability Act (HIPAA). Advances in the private cloud space are beginning to address some of these issues. Technologists are experimenting with container and enclave technologies, but the main issues with private cloud use in this context continue to be data security and costs (ingress/egress, computation on large-scale analysis).

In order to provide accurate and timely care tailored to the individual level, there is much work to be done in standardizing data collection, input methods, coding, storage, access, and analysis. Medical data is increasingly being stored in Electronic Health Records (EHRs), but not all providers have a common format for data, limiting the ability to use common analysis tools across various electronic data warehouses. Additionally, healthcare information varies in nature and can be difficult to collect and input into existing systems (e.g., symptoms, interactions, lab reports, and photographs). The systems may not code these datasets correctly, resulting in unstructured data in the record. Unstructured data, such as notes, contain valuable care information but that information is difficult to extract. This limits the ability for machine understanding and analysis. Examples of projects that utilize natural language processing to convert unstructured data into structured data were discussed, but this is still an evolving process.

Data structure standards and easier access to reliable raw data were important topics of discussion during the workshop. Many arguments for developing an accepted standard for data structure were raised. According to the National Human Genome Research Institute, a division of the National Institutes of Health, the human genome consists of about three billion base pairs [6] but about 99.5% of our DNA is the same as all other humans [7]. A standard method of separating out the ~1% of the human genome variances would significantly reduce the data size and help simplify management, transfer, and storage constraints between collaborators and analysis sites. Standardizing data structure would help prevent scientists from spending more time in going back to raw data for analysis, in addition to increasing the opportunities for machine learning and cross-provider databases. However, it should be understood that going back to raw data is necessary in some cases, relative to what the original research question was when the raw data/metadata was generated during sequencing. Particular data structures could act as a barrier to other scientists when looking at the structured data as opposed to the raw data. This is particularly true when scientists from different focus areas wish to compare datasets to identify similarities or differences between organisms (i.e., comparing a human genome to a particular plant genome). To this point, workshop discussions repeatedly highlighted the need for easier community access to reliable raw data and metadata of previously sequenced samples.

### Metagenomics

Metagenomics is defined as the direct genetic analysis of genomes contained within an environmental sample [8] and applies to any category of organisms. The study of metagenomics has provided critical insight into understanding the function and relationships of the human body and the world we live in over the past several years, and is becoming the focus of an increasing number of research projects and scientific organizations. One such project is the White House Microbiome Initiative, announced in May 2016. While the initiative was announced after this workshop was held, it highlights the importance of expanding this field of study. This White House Initiative has three specific goals, developed throughout the course of a year-long fact-finding process. These goals are:

1. Supporting interdisciplinary research to answer fundamental questions about microbiomes in diverse ecosystems.
2. Developing platform technologies that will generate insights and help share knowledge of microbiomes in diverse ecosystems and enhance access to microbiome data.
3. Expanding the microbiome workforce through citizen science and educational opportunities [9].

The increasing focus on the field of metagenomics, combined with rapid advances in technology, are creating massive amounts of data at rates that are difficult to predict. Therefore, close coordination and regular dialogue between research scientists, technology experts, and policymakers are crucial.

Understanding the underlying functions, relationships, and interactions between various parts of the same organism and between organisms, is not only reliant on significant computational power, but the ability to share datasets with other scientists to benefit from their expertise as well. This happens not only within the same discipline, but across disciplines. Examples were presented in the workshop that showed how cross-disciplinary studies could lead to the creation of extensive and powerful systems biology models. Intersections between such models can be found at great evolutionary distances, including between trees and humans, thus demonstrating the strongly conserved nature of many basic biological functions [10]. Researchers are also studying microbiomes in the earth’s atmosphere and how their distribution patterns relate to climate patterns. Big data approaches are fundamentally changing the way that biological research is being performed. As such, the need for data access, data repositories, and means with which to do rapid, large-scale bulk transfers was quite clear.

Many examples of scientific discoveries that were a result of sharing datasets between researchers and laboratories were discussed. However, two primary barriers currently exist that prevent scientists from using another’s dataset on a larger scale: not being able to locate targeted datasets in a timely fashion and the time required to verify the data’s quality and integrity. The combination of these issues often results in a researcher starting from scratch to sequence their own samples, made easier because of the prevalence of low-cost sequencers. This cycle compounds the problem of scattered datasets for further analysis. Still, it was noted that structured databases do exist and are heavily utilized in the community. The Department of Energy’s Joint Genome Institute (JGI) was founded in 1997 and has been a leader in large-scale, sequence-based science ever since. External data sources play a key role in metagenomics efforts at JGI. The sources come from external collaborators and community archives, such as the National Center for Biotechnology Information (NCBI) short read archive. Biological data made available by JGI, NCBI, The European Bioinformatics Institute (EMBL-EBI) and many other data repositories are invaluable resources and their best practices and lessons learned should be used as a baseline in the development of additional databases that will serve the genomics community.

## Workshop description and structure

The Cross Connects Workshop series brings together professionals at the intersection of scientific research, experimental facilities and cyberinfrastructure. The fourth workshop in this series was held at Lawrence Berkeley Lab in April 2016 with a focus on Improving Data Mobility & Management for Bioinformatics. Only by bringing these three groups of experts in computing, bioinformatics, and networking together can complex, end-to-end data mobility and management challenges be discussed and addressed. Table 1 lists the workshop speakers and presentation titles.  A full agenda for the meeting and links to the presentations can be found on the Workshop web pages [11–13].

**Table 1:** Workshop speakers and presentation titles

Speaker	Presentation Title
Inder Monga, ESnet/LBNL	*Welcome & Workshop Kickoff*
William Barnett, CTSI and Regenstrief Institute, Indiana University	*The Promise of Precision Medicine*
Sean Mooney, UW Medicine; BIME, University of Washington	*Data Driven Translation of Research to Enable Precision Medicine*
Robert Freimuth, Mayo Clinic	*Genomic-based Precision Medicine at Scale*
Chris Bradburne, Johns Hopkins University, Applied Physics Lab	*Precision Medicine and Exposomics in the Military*
Larry Smarr, Calit2	*Analyzing the Human Gut Microbiome Dynamics in Health and Disease Using Supercomputers and Supernetworks*
Joe Hesse, UCSF	*Securing and Auditing HPC Workloads for Human Subjects’ Protection and HIPAA Compliance*
Peter Denes & Kristofer Bouchard, National Center for Electron Microscopy	*It Takes Big Data to See Small Things*
Dan Jacobson, ORNL Biosciences Division	*Data Challenges at the Intersection of Human and Plant Biome Discovery and Analysis*
Zaid Abdo, Colorado State	*The Study of Complex Systems*
Natalia Ivanova, JGI	*Data challenges in distributed microbiome research*
Steven Newhouse, EMBL-EBI	*Managing reference data sets within Europe*
Kjiersten Fagnan, NERSC/JGI	*Computational Demands of the DOE Joint Genome Institute (JGI) Metagenome Program*
Eli Dart, ESnet/LBNL	*The Medical Science DMZ*
Ravi Madduri, Globus	*Managing Big Biomedical Data Using Globus*

The workshop was organized by leaders in the field (see Appendix 1 for the program committee), with the goal of having an interactive meeting. The workshop brought together more than 50 members of the community in person and a small number of remote participants viewed the live stream of the event (See Appendix 2 for a full list of participants).

## Findings and recommendations

The workshop discussions covered a wide variety of topics of both immediate and strategic importance in Precision Medicine and Metagenomics. The overarching issue facing both fields is data growth: how best to manage it, store it, and share it. Both fields are wholly dependent on computation, and breakthroughs are accelerated through collaboration. Several key findings are summarized below, followed by some recommendations and future considerations.

### Findings

1. There is a significant need for a series of easily accessible datastores that adhere to community-determined quality standards to hold raw genomics data and metadata.
  - Currently, resequencing genome data is cheaper than storing the raw (image-based) data over long periods of time. Microbiome datasets represent a snapshot of the species present in a microbial community in a specific location at a given moment in time. As the composition of each community will change over time and in response to environmental perturbations, the same site may be sequenced multiple times. The need for easily accessible repositories of raw metagenomic data (reads) and metadata was a common topic of discussion throughout the workshop. While many datasets exist today, the process of locating and then verifying a specific dataset’s quality are major barriers, slowing the progress of discovery. Given the rapid growth in size, raw data and metadata will likely need to be stored in a series of accessible datastores that require shared datasets to adhere to resource-specific quality standards determined by the community.
  - ESnet is looking into the possibility of partnering with science and computing stakeholders, such as JGI and the Oak Ridge National Laboratory to lead the investigation of such an effort, including possible funding sources and technical architectures. ESnet can play a very valuable role within the DOE system both as a transport layer, coordinator, and as a neutral broker between national labs, other government agencies, and the university community. Discussions highlighted the need to develop compute, analysis, and transfer workflows tailored to the biosciences community due to the rapid advances in genomic technology. Larry Smarr of the University of California, San Diego and Principal Investigator of the National Science Foundation-funded Pacific Research Platform (PRP) [14], volunteered the PRP to enable and support these collaborations to document the use cases for the development of biosciences-specific workflows at scale.
2. Additional coordination and work is needed to overcome barriers associated with privacy policy compliance and “data hoarding.” Two main barriers to open data exchanges were discussed at the workshop: the compliance with policies around PHI, HIPAA and FISMA guidelines using current infrastructures, and “data hoarding.”
  - Addressing the compliance issues will require additional coordination between scientists, cloud providers, facility personnel, and policymakers to ensure policy compliance is possible without sacrificing data analysis in a collaborative, geographically-dispersed environment.
  - Data hoarding is somewhat alleviated by grant awards and publications being contingent on actionable data management policies, but is difficult to resolve when the hoarding period exists between data collection and publication. This can be a significant amount of time, in which the relevancy of data can change.
3. The “Medical Science DMZ” is a design pattern that can support workflows containing PHI.
  - The Science DMZ [15] was discussed as a proven design pattern for accelerating science flows among facilities. With some modification, this pattern can also be applied to bioinformatics with an emphasis on supporting workflows containing PHI. ESnet currently maintains a robust knowledge base for optimizing systems and networks for data-intensive scientific workflows.
  - It was suggested that community members collaborate on creating another easily accessible resource, but specifically written for the bioinformatics community to support policy-compliant infrastructure models, data transfers, system tuning, data analysis, data standards, etc. Multiple experts in the networking and security fields collaborated on a Medical Science DMZ paper, published after the workshop in October 2017 [16]. Efforts to identify a small number of pilot sites to test the viability of the determined approach will likely follow the publication.
4. The JGI Archive and Metadata Organizer (JAMO) is a notable example for data curation and metadata tagging for dissemination. The JGI Archive and Metadata Organizer (JAMO), launched in 2013, was presented as a good model for data curation and metadata tagging for dissemination in workshop discussions. It was suggested that JAMO developers share best practices to help others in the community implement this model.
5. New software and portals are needed to efficiently manage and serve bioinformatics data. There is an increasing need for sustainable, well maintained, quality software and portals built for the management, access, and mobility of bioinformatics data. This is a complicated problem and will require much more than an increase in funding from federal (and international) funding agencies. Solutions will need to involve coordination between scientific and clinical researchers, private companies, funding agencies, infrastructure facilities, and educators.

### Recommendations

1. Scientists and researchers need to be able to easily locate and verify the quality and compliance of (meta)genomic raw data and metadata. A large-scale solution toward data storage and standards is needed in order to accelerate scientific discovery in bioinformatics (see finding 1).
2. Use cases for the development of bio-specific workflows at scale should be organized and documented using the PRP participants and partners (see finding 2).
3. DOE institutions, such as ESnet in partnership with national labs, and other members of the community should develop a bioinformatics-specific community resource of best practices and recommendations for policy-compliant infrastructure models, data transfers, system tuning, data analysis, data standards, etc. (see findings 4,5).
4. At the conclusion of the workshop and through post-workshop attendee feedback, it was clear that the topic of data movement and collaboration is critical to the field, but issues around data privacy, policy compliance, and the lack of universally accepted data structure is currently a larger hindrance to data movement than physical infrastructure in some cases. Community-wide efforts to address these issues are needed in parallel with ongoing national initiatives that are shaping the future of computational and network facilities for bioinformatics.

## Appendix 1 Workshop Planning Committee

The workshop was organized by a team of researchers who met on a weekly basis, identified program topics and speakers, invited participants, and helped run the meeting. This committee consisted of:

William Barnett - Indiana University (Precision Medicine Program Chair).

Brooklin Gore - ESnet, Lawrence Berkeley National Laboratory.

Mary Hester – ESnet, Lawrence Berkeley National Laboratory.

Dan Jacobson - Oak Ridge National Laboratory (Metagenomics Program Chair).

Nikos Kyrpides - Lawrence Berkeley National Laboratory, JGI.

Kathryn Petersen Mace – ESnet, Lawrence Berkeley National Laboratory.

Ravi Madduri - Argonne National Laboratory.

Inder Monga – ESnet, Lawrence Berkeley National Laboratory.

Predrag Radulovic - Indiana University.

Lauren Rotman – ESnet, Lawrence Berkeley National Laboratory.

Jennifer Schopf - Indiana University

## Appendix 2 Participants

59 participants from 33 institutions took part in the two-day meeting.

**Table:**

Zaid Abdo, Colorado State University.
Gladys Andino, Purdue University
Mark Arick II, Mississippi State
Bill Barnett, Indiana CTSI
Kristofer Bouchard, LBNL
Chris Bradburne, John Hopkins University
Joe Breen, University of Utah
Anushka Brownley, Bio Team
Jun Cao, Dow AgroSciences
Travis Cotton, Texas Tech University
Eli Dart, ESnet / LBNL
Peter Denes, LBNL
Kjiersten Fagnan, LBNL / JGI
Louis Fox, CENIC
Robert Freimuth, Mayo Clinic
Cinta Gomez, LBNL
Brooklin Gore, ESnet / LBNL
Kyle Halliday, LLNL
Joe Hesse, UCSF
Mary Hester, ESnet / LBNL
David Hiatt, HGST
Natailia Ivanova, JGI
Daniel Jacobson, ORNL
Piet Jones, ORNL
Kristy Kallback-Rose, Indiana University
Mohammad Asif Khan, Perdana University
Patrick Leyshock, Oregon Health & Science University
Sam Liston, University of Utah
Susan Lucas, ESnet / LBNL
Kathryn Petersen Mace, ESnet / LBNL
Ravi Madduri, ANL
Jeffrey Mast, Teres Tech
Ramil Mauleon, International Rice Research Institute
Erik McCroskey, UC Berkeley
Xiandong Meng, JGI
Inder Monga, ESnet / LBNL
Sean Mooney, University of Washington
Steve Newhouse, EBI
Peter Nugent, LBNL
Predrag Radulovic, Indiana University
Phil Reese, Stanford
Alex Ropelewski, Pittsburgh Supercomputing Center
Lauren Rotman, ESnet / LBNL
Patrick Schmitz, UC Berkeley
Jennifer Schopf, Indiana University
Asya Shklyar, Bio Team
Muhammad Farhan Sjaugi, Perdana University
Larry Smarr, UCSD
Cory Snavely, LBNL
Josh Sonstroem, UCSC
Melissa Stockman, ESnet / LBNL
Rune Stromsness, LBNL
Michael Sullivan, Internet2
Xiangying Sun, Purdue University
Kevin Thompson, NSF
Brian Tierney, ESnet/ LBNL
Le Yan, LSU
Jason Zurawski, ESnet / LBNL
Peter Zwart, LBNL

## Abbreviations

BIME: Biomedical Informatics and Medical Education, located at the University of Washington, United States
CTSI: Indiana Clinical and Translational Sciences Institute, a statewide collaboration between Indiana University, Purdue University, and the University of Notre Dame, Indiana, United States
DHHS: Department of Health and Human Services
DNA: Deoxyribonucleic acid
DOE: Department of Energy
EHR: Electronic Health Record
EMBL-EBI: European Bioinformatics Institute
FISMA: The Federal Information Security Management Act
HIPAA: Health Insurance Portability and Accountability Act
JAMO: JGI Archive and Metadata Organizer
JGI: Joint Genome Institute
LBNL: Lawrence Berkeley National Laboratory
NCBI: National Center for Biotechnology Information
NERSC: National Energy Research Scientific Computing Center
NHGRI: National Human Genome Research Institute
ORNL: Oak Ridge National Laboratory
PHI: Protected Health Information
PRP: Pacific Research Platform
RNA: Ribonucleic acid
Science DMZ: A network design pattern that is designed for data-intensive science/research network traffic. It is a portion of the computer network that is built at or near the campus or laboratory’s local network perimeter that is designed such that equipment, configuration, and security policies are optimized for high-performance scientific applications
UCSF: University of California, San Francisco
UW: University of Washington
